# Supplementary material for: Uncertainty-inspired open set learning for retinal anomaly identification
Source: Nat Commun. 2023 Oct 24;14:6757. doi: 10.1038/s41467-023-42444-7 (PMC10598011; doi:10.1038/s41467-023-42444-7)
Supplement: Supplementary file 1 — Supplementary Information [file 41467_2023_42444_MOESM1_ESM.pdf]

Supplementary Information

Supplementary Table 1: Precision of different models on different testing sets (%)

Category	Internal testing set			TC-JSIEC			TC-unseen		
	Standard AI model	UIOS	UIOS + Thresholding	Standard AI model	UIOS	UIOS + Thresholding	Standard AI model	UIOS	UIOS + Thresholding
Normal	95.08	98.38	99.76	69.05	77.78	81.82	62.49	76.77	87.10
TF	95.60	94.62	98.68	68.75	65.00	90.00	74.33	81.72	95.90
PM	95.43	99.42	100.00	98.18	100.00	100.00	82.50	81.16	98.31
GL	100.00	99.50	100.00	70.00	88.89	100.00	76.49	75.05	92.96
RVO	96.85	95.56	99.21	100.00	100.00	100.00	92.46	90.37	99.49
RD	88.24	98.91	100.00	98.21	100.00	100.00	35.89	69.32	91.74
AMD	96.30	96.91	99.21	73.96	88.10	98.63	53.67	57.71	74.07
DR	95.30	98.48	99.62	93.98	95.56	100.00	65.06	85.21	96.89
CSCR	68.22	96.34	100.00	51.85	63.64	100.00	76.05	82.08	95.56
Average	92.34	97.57	99.61	80.44	86.55	96.72	68.77	77.71	92.45

Supplementary Table 2: Sensitivity of different models on different testing sets (%)

Category	Internal testing set			TC-JSIEC			TC-unseen		
	Standard AI model	UIOS	UIOS + Thresholding	Standard AI model	UIOS	UIOS + Thresholding	Standard AI model	UIOS	UIOS + Thresholding
Normal	100.00	100.00	100.00	76.32	92.11	100.00	95.01	90.73	99.43
TF	90.63	91.67	98.68	84.62	100.00	100.00	49.40	75.40	83.27
PM	96.53	98.27	98.80	100.00	100.00	100.00	77.46	78.87	91.34
GL	94.66	97.57	100.00	53.85	61.54	87.50	78.93	81.91	97.29
RVO	94.62	99.23	100.00	75.76	90.91	100.00	50.69	80.17	94.69
RD	99.26	99.63	100.00	96.49	89.47	97.73	76.95	75.31	93.46
AMD	80.97	97.58	99.60	95.95	100.00	100.00	35.56	44.38	79.37
DR	91.29	97.60	99.62	73.58	81.13	93.85	45.33	81.31	95.20
CSCR	84.88	91.86	98.67	100.00	100.00	100.00	83.60	85.68	90.80
Average	92.54	97.04	99.49	84.06	90.57	97.67	65.88	77.08	91.65

Supplementary Table 3: Specificity of different models on different testing sets (%)

Category	Internal testing set			TC-JSIEC			TC-unseen		
	Standard AI model	UIOS	UIOS + Thresholding	Standard AI model	UIOS	UIOS + Thresholding	Standard AI model	UIOS	UIOS + Thresholding
Normal	98.61	99.56	99.93	96.73	97.48	98.73	89.86	95.12	96.74
TF	99.79	99.74	99.94	98.82	98.34	99.69	97.32	97.35	99.40
PM	99.56	99.95	100.00	99.74	100.00	100.00	99.00	98.89	99.89
GL	100.00	99.94	100.00	99.29	99.76	100.00	96.20	95.74	98.88
RVO	99.79	99.68	99.94	100.00	100.00	100.00	99.55	99.08	99.94
RD	97.93	99.83	100.00	99.74	100.00	100.00	90.38	97.67	99.51
AMD	99.48	99.48	99.87	93.07	97.23	99.62	97.02	96.84	98.08
DR	99.11	99.70	99.94	98.48	98.78	100.00	95.62	97.46	99.59
CSCR	98.23	99.84	100.00	96.91	98.10	100.00	96.53	97.53	99.35
Average	99.17	99.75	99.96	98.09	98.86	99.78	95.72	97.30	99.04

Supplementary Table 4: Distribution of data after filtering out samples with uncertainty scores above the threshold value of θ

Dataset	Internal testing set		TC-JSIEC		TC-unseen	
	Original	After thresholding	Original	After thresholding	Original	After thresholding
Normal	425	423	38	18	561	353
TF	96	76	13	9	504	281
PM	173	166	54	53	213	127
GL	206	183	13	8	503	258
RVO	130	126	66	51	363	207
RD	272	252	57	44	243	107
AMD	289	252	74	72	329	126
DR	333	261	106	65	567	229
CSCR	86	75	14	14	433	261
Total	2,010	1,814	435	334	3,716	1,949

Supplementary Table 4 shows the distribution of different testing sets after filtering out samples with uncertainty scores above the threshold value of θ. As shown in Supplementary Table 4, most of the samples in the internal testing set, which have a similar feature distribution to the training data, obtained high-confidence prediction results. However, the two external test data sets, TC-JSIEC and TC-unseen, have a large difference in feature distribution from the training data. Consequently, more samples from these sets required double-checking by the ophthalmologist to avoid mis-/under-diagnosis may be caused by the samples with low confidence prediction results. These results are consistent with the observation in clinical practice that junior physicians can accurately identify fundus diseases with distinctive features with high confidence. However, data with ambiguous features are often judged with low confidence, and it is necessary to seek further confirmation from a senior ophthalmologist before a final diagnosis can be made.

Supplementary Table 5: F1 scores of different methods on internal testing set (%)

Category	MC-Drop	Ensemble	TTA	Entropy	UIOS
Normal	99.05	97.69	97.79	97.67	99.18
TF	95.43	94.18	90.00	91.30	93.12
PM	91.54	97.66	95.51	95.18	98.84
GL	91.31	93.58	92.52	93.09	98.53
RVO	93.63	96.44	96.53	96.12	97.36
RD	95.64	96.38	92.51	92.13	99.27
AMD	90.65	90.98	85.77	85.60	97.24
DR	95.20	95.41	91.56	91.04	98.04
CSCR	87.06	89.02	82.29	81.03	94.05
Average	93.28	94.59	91.61	91.46	97.29

Supplementary Table 6: F1 scores of different methods on internal testing set after thresholding (%)

Category	MC-Drop	Ensemble	TTA	Entropy	UIOS
Normal	99.87	99.37	99.60	99.88	99.88
TF	97.56	98.59	96.92	98.99	98.68
PM	97.44	99.66	97.58	100.00	99.39
GL	97.71	99.49	97.17	99.73	100.00
RVO	99.07	98.36	98.06	99.53	99.60
RD	99.40	99.18	97.05	99.38	100.00
AMD	97.23	98.98	92.45	98.54	99.41
DR	98.84	98.87	96.72	99.44	99.62
CSCR	98.97	95.45	90.43	96.00	99.33
Average	98.45	98.66	96.22	99.05	99.55

Supplementary Table 7: P-values of F1 scores for UIOS model compared to other methods on different datasets

Methods	Internal testing set	TC-JSIEC	TC-unseen	Internal testing set+thresholding	TC-JSIEC+thresholding	TC-unseen dataset+thresholding
UIOS->Baseline	**0.029**	**0.006**	**0.008**	/	/	/
UIOS->MC-Drop	**0.008**	**0.026**	**0.001**	**0.005**	0.091	**0.004**
UIOS->Ensemble	**0.009**	0.376	**0.001**	0.058	0.286	**0.009**
UIOS->Entropy	**0.004**	**0.045**	**0.001**	0.235	**0.046**	**0.027**
UIOS->TTA	**0.003**	**0.030**	**0.001**	**0.007**	**0.042**	**0.0002**

P-Value was calculated by two-sided T-Test, and no adjustments were made for multiple comparisons.

Supplementary Table 8: P-values of AUC for UIOS model compared to other methods on different datasets

Methods	Internal testing set	TC-JSIEC	TC-unseen	Internal testing set+thresholding	TC-JSIEC+thresholding	TC-unseen dataset+thresholding
UIOS->Baseline	0.371	**0.002**	0.196	/	/	/
UIOS->MC-Drop	**0.036**	0.055	**0.003**	0.058	0.115	**0.006**
UIOS->Ensemble	**0.036**	0.219	**0.036**	0.071	0.276	**0.033**
UIOS->Entropy	0.565	**0.020**	0.610	0.263	**0.029**	0.259
UIOS->TTA	**0.032**	**0.023**	**0.010**	**0.017**	**0.015**	**0.005**

P-Value was calculated by two-sided T-Test, and no adjustments were made for multiple comparisons.

Supplementary Table 9: Rates for prompting a human grading and correct disease prediction with high uncertainty above threshold

Category	Internal testing set (%)	TC-JSIEC(%)	TC-unseen dataset (%)
Rate for prompting a human grading	9.75	23.22	47.55
Rate of correct disease prediction with high uncertainty above threshold	8.06	15.40	30.09

Supplementary Table 10: F1 scores of different methods on TC-JSIEC set (%)

Category	MC-Drop	Ensemble	TTA	Entropy	UIOS
Normal	34.78	67.53	57.14	58.06	84.34
TF	72.73	81.25	81.48	85.71	78.79
PM	97.20	99.08	94.64	94.64	100.00
GL	60.87	80.00	56.00	56.00	72.73
RVO	84.75	89.08	90.32	90.32	95.24
RD	92.45	96.36	91.43	91.43	94.44
AMD	74.00	93.08	87.50	87.50	93.67
DR	86.01	88.44	86.73	86.73	87.76
CSCR	59.09	70.00	48.28	48.28	77.78
Average	73.54	84.98	77.06	77.63	87.19

Supplementary Table 11: F1 scores of different methods on TC-unseen dataset (%)

Category	MC-Drop	Ensemble	TTA	Entropy	UIOS
Normal	65.97	72.54	74.91	74.21	83.17
TF	55.43	49.94	55.67	54.17	78.43
PM	45.36	72.22	66.24	65.38	80.00
GL	69.00	73.00	74.20	74.37	78.33
RVO	67.01	60.23	65.34	65.21	84.96
RD	61.28	60.68	60.64	61.84	72.19
AMD	47.72	44.05	39.06	39.14	50.17
DR	72.00	69.68	79.28	79.47	83.21
CSCR	73.99	74.53	75.35	74.84	83.84
Average	61.97	64.10	65.63	65.40	77.15

Supplementary Table 12: F1 scores of different methods on TC-JSIEC set after thresholding (%)

Category	MC-Drop	Ensemble	TTA	Entropy	UIOS
Normal	22.22	66.67	63.64	40.00	90.00
TF	92.31	100.00	100.00	50.00	94.74
PM	100.00	100.00	96.91	99.05	100.00
GL	90.91	100.00	76.92	66.67	93.33
RVO	91.30	91.18	95.15	95.83	100.00
RD	100.00	100.00	93.33	98.36	98.85
AMD	85.31	96.97	95.33	95.74	99.31
DR	89.71	98.63	91.34	98.46	96.83
CSCR	76.19	85.71	61.54	88.00	100.00
Average	83.11	93.24	86.02	81.35	97.01

Supplementary Table 13: F1 scores of different methods on TC-unseen dataset after thresholding (%)

Category	MC-Drop	Ensemble	TTA	Entropy	UIOS
Normal	82.89	78.15	84.64	87.68	92.86
TF	72.89	55.59	58.48	55.46	89.14
PM	45.28	84.54	77.08	88.24	94.69
GL	84.40	92.60	83.29	94.31	95.08
RVO	74.56	63.89	68.92	80.19	97.03
RD	81.40	84.48	65.95	85.11	92.59
AMD	58.71	73.91	48.00	70.00	76.63
DR	81.91	80.31	84.33	93.46	96.04
CSCR	87.92	89.81	80.88	89.49	93.12
Average	74.44	78.14	72.40	82.66	91.91

Supplementary Table 14: The abnormal detection rates of different methods on different datasets (%)

Methods	NTC	NTC-JSIEC	Low-quality	RETOUCH	OCTA	VOC 2012	Time (ms/per image)
MC-Drop	71.96	69.92	54.32	1.40	59.21	73.56	18.11
Ensemble	79.20	81.08	71.11	5.49	**100.00**	83.85	1.01
Entropy	82.61	**83.27**	84.62	2.72	0.00	44.85	**0.34**
TTA	56.30	58.37	55.63	26.10	2.96	48.90	4.87
UIOS	**86.67**	82.27	**89.40**	**99.81**	99.01	**96.18**	**0.34**

Supplementary Table 15: Sample size of the target categories datasets (%)

Dataset	Primary TC dataset				TC-unseen	TC-JSIEC
	Total	Training set	Validation set	Testing set		
Normal	2,125	1,275	425	425	561	38
TF	478	286	96	96	504	13
PM	863	517	173	173	213	54
GL	1,026	615	205	206	503	13
RVO	650	390	130	130	363	66
RD	1,359	815	272	272	243	57
AMD	1,443	865	289	289	329	74
DR	1,661	996	332	333	567	106
CSCR	429	257	86	86	433	14
Total	10,034	6,016	2,008	2,010	3,716	435

Supplementary Table 16. Inclusion criteria for Target Categories (TC) retinal diseases

**Categories**	Primary TC dataset	**TC-unseen dataset**
Normal	Orange-red fundus without any pathological changes.	Orange-red fundus without pathological changes, but maybe with indistinct C/D ratio, blurring boundary of optic disc, few exposed choroidal large vessels, overexposure or underexposure, suspicious lens stains, or generally slightly blur due to medium opacity or defocus.
Tigroid Fundus (TF)	Extensive/diffuse attenuation of RPE exposed the underlying large choroidal vessels, with an area larger than half field.	Local attenuation of the RPE with visibility of underlying regional choroidal vessels with an area less than half field.
Pathological Myopia (PM)	Extensive tigroid fundus with massive chorioretinal atrophy, Fuchs spot, lacquer cracks, CNV, subretinal hemorrhage.	Obvious tigroid fundus with titled optic disc, optic disc arc atrophy, choroid thinning, but without massive focal chorioretinal atrophy or macular lesions. Or combined with epiretinal membrane and retinal holes leading to retinal detachment,
Glaucoma (GL)	Vertical C/D ratio $\geq$0.6, cup excavation and pale, thinning of neuroretinal rim, notching and bayoneting of vessels, baring of circumlinear blood vessels, laminar dot sign, disc hemorrhages, RNFL defects, peripapillary atrophy.	Enlarged C/D ratio without other classic glaucomatous damages of optic head, may with tilt, neovascularization or overexposure of optic disc. May combined with other lesions like tigroid fundus, drusen, hemorrhage, etc.
Retinal Vein Occlusion (RVO)	Tortuosity and dilatation of affected branches of veins, with variable degrees of intraretinal hemorrhage (dot-, blot- or flame-like), cotton wool spots, hard exudates, macular edema or subretinal fluid in the distribution of affected veins.	Sheathing, sclerosis or slightly dilation of affected veins, diffused/local distribution of variable hemorrhage but not strictly accompanying veins, maybe with chronic macular oedema, collateral vessels, glaucomatous optic nerve changes, retinal neovascularization, vitreous/preretinal hemorrhage or tractional retinal detachment. Laser spots may be seen.
Retinal Detachment (RD)	Detaching retina layer with a convex configuration and corrugated appearance, maybe with variable retinal breaks in view	Only small part of ambiguous detaching retina in view, or combined with other pathological lesions like massive vitreous hemorrhage, proliferative vitreoretinopathy or chorioretinal atrophy.
Age-Related Macular Degeneration (AMD)	Multiple dense or confluent drusen, focal hyper- and/or hypopigmentation of the RPE, thinning or geographic atrophy of RPE, choroidal neovascularization leading to fibrovascular/serous PED, sub-foveal atrophy or fibrosis secondary to an RPE tear	Only small or intermediate-sized drusen without other lesions, or orange-reddish bulb-like lesions associated with significant hemorrhagic and exudative detachments of retina and retinal pigment epithelium and hard exudates in polypoidal choroidal vasculopathy.
Diabetic Retinopathy (DR)	Multiple microaneurysms, variable dot/blot-like hemorrhages, hard exudates, maybe with macular oedema, neovascularization, vitreous/preretinal hemorrhage or preretinal proliferative membrane.	Only microaneurysms (Mild NPDR), or severe proliferative membrane and vitreous hemorrhage covering the retinal characteristics. Any stages with laser spots.
Central Serous Chorioretinopathy (CSCR)	Round or oval macular retinal elevation with distinct margins and turbid fluid underneath, small and yellow sub-retinal deposits. May with depigmented RPE foci or small patches of RPE atrophy or hyperplasia.	Ambiguous retinal elevation with indistinct margins, or liquid partially absorbed leaving macular RPE mottling.

Supplementary Table 17. Diagnosis and numbers of images in non-target categories (NTC) dataset and NTC-JSIEC dataset

Datasets	number
NTC dataset	1380
Retinal Artery Occlusion	183
Macular Hole	265
Epiretinal Membrane	301
Vogt-Koyanagi-Harada Disease	264
Retinitis Pigmentosa	308
Asteroid Hyalosis	59
NTC-JSIEC dataset	502
Retinal Artery Occlusion	16
Macular Hole	23
Epiretinal membrane	26
Vogt-Koyanagi-Harada Disease	14
Retinitis pigmentosa	22
Asteroid hyalosis	14
Optic atrophy	12
Hypertensive retinopathy	15
Large optic cup	50
Bietti crystalline dystrophy	8
Disc swelling and elevation	13
Dragged disc	10
Congenital disc abnormality	10
Peripheral retinal degeneration and breaks	14
Myelinated nerve fiber	11
Fundus neoplasm	8
Yellow-white spots	29
Vessel tortuosity	14
Chorioretinal atrophy-coloboma	15
Silicon oil in eye	19
Blur fundus	159

Supplementary Table 18: F1 scores (%) of the ablation experiments

Backbone	L_{UN}	L_{TUN}	Internal testing set	TC-JSIEC	TC-unseen	Average
√	×	×	92.20	80.69	64.74	79.21
√	√	×	94.02	76.05	64.72	78.27
√	×	√	**97.29**	**87.19**	**77.15**	**87.21**

We conduct ablation experiments to demonstrate the effectiveness of the main components in our proposed UIOS. Supplementary Table 18 shows the ablation results. In our study, the pre-trained ResNet-50 is employed as our backbone for capturing the feature information in fundus images, Backbone+L_{UN} indicates the combination of ResNet-50 and subjective logical (SL) evidential uncertainty theory, while Backbone+L_{TUN} represent our proposed UIOS method. As shown in Supplementary Table 18, compared to the Backbone, Backbone+ L_{UN} to enable the model to generate the prediction with uncertainty score based on the features that were parameterized by Dirichlet concentration. However, as shown in Supplementary Table 18, the F1 score of Backbone+L_{UN} on most testing sets is lower than that of Backbone, mainly because Dirichlet re-parameterization changes the original feature distribution, reducing the model's confidence in the class-related evidence, thus leading to lower performance. Focusing on this problem, we further improved the loss function by introducing a temperature cross-entropy loss function, which can enhance the model's confidence in the features that are re-parameterized by Dirichlet, thereby improving the performance in detecting retinal fundus diseases. Thus, it can be seen from Supplementary Table 18 that our proposed UIOS (Backbone+L_{TUN}) achieves the highest performance compared to Backbone and Backbone+L_{UN} on the internal testing set, and two external test sets, the CJSIEC dataset and Non-typical CRD set, both of which have significantly different feature distributions from the training data. The F1 score of our UIOS on three testing set reaches 97.29%, 87.19%, and 77.15%, respectively. These experimental results further demonstrate the effectiveness of our proposed UIOS.

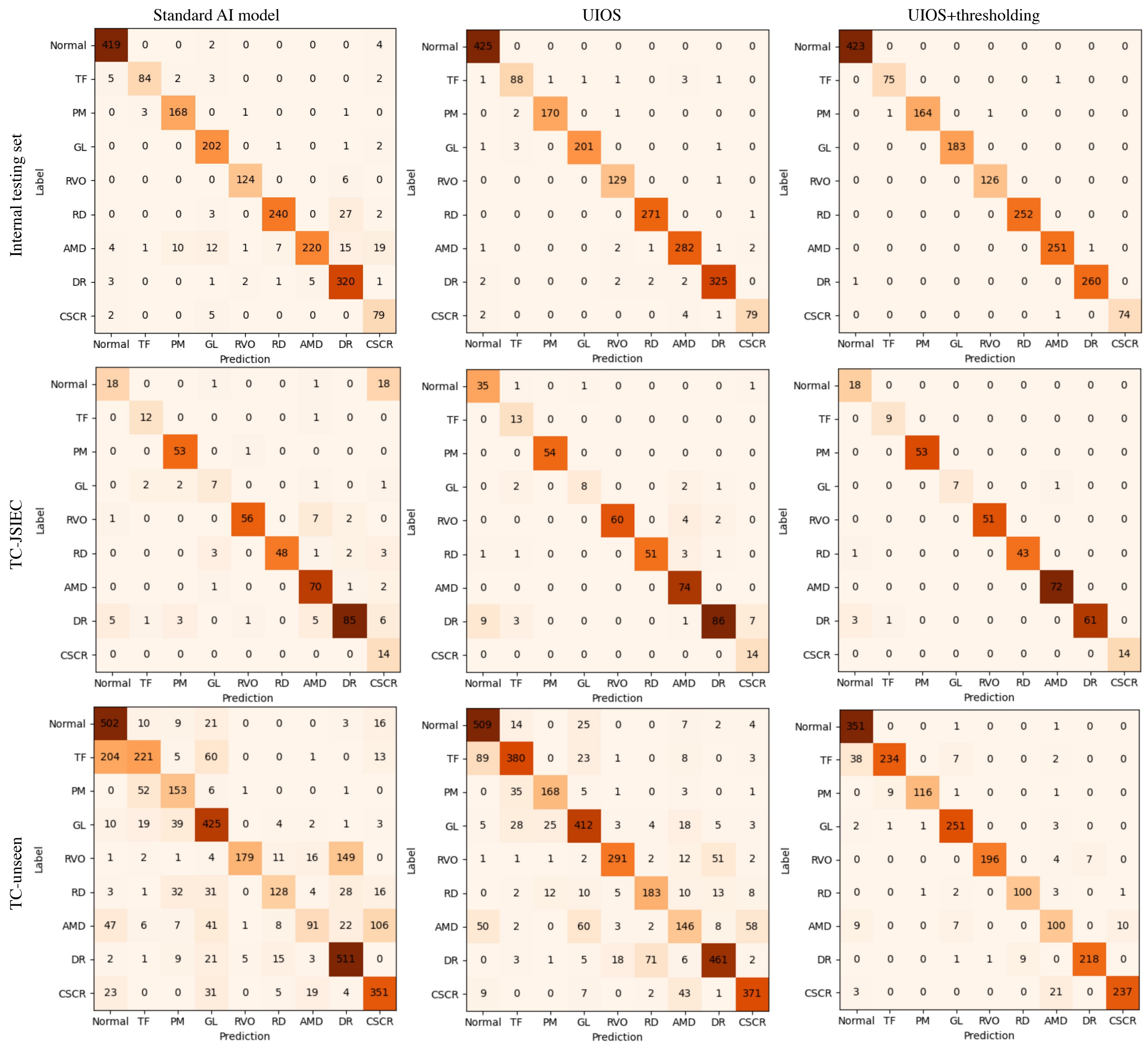

Supplementary Fig. 1. The confusion matrix of the standard AI model, our UIOS, and UIOS+Thresholding in internal and external testing datasets

As shown in Supplementary Fig. 1, our UIOS outperformed the standard AI model in terms of confusion matrix for all test sets. Furthermore, when applying our thresholding strategy (UIOS+thresholding) to suggest that samples with uncertainty scores above the threshold seek manual check by an ophthalmologist, we observed a further significant improvement in the confusion matrix and a significant reduction in misclassified samples.

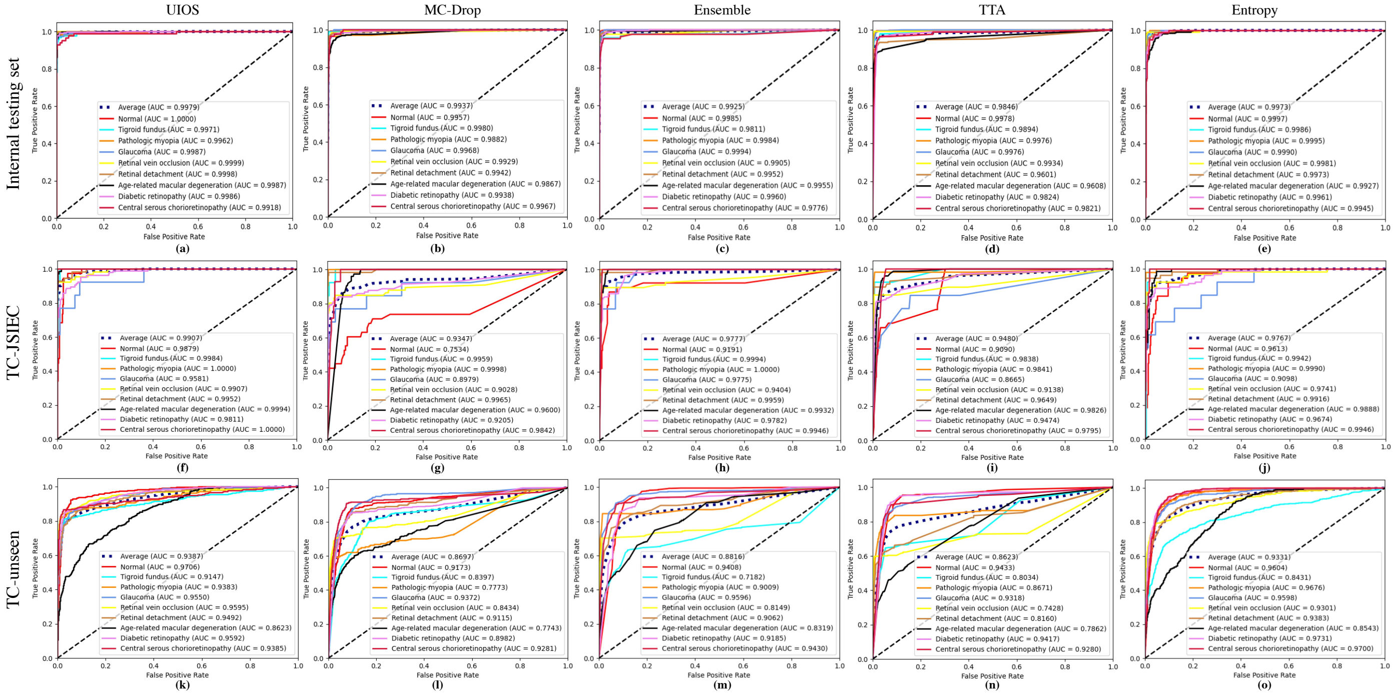

Supplementary Fig. 2. The receiver operating characteristic (ROC) curves of our UIOS model and other uncertainty-based methods in internal and two external testing datasets. Source data are provided as a Source Data file.

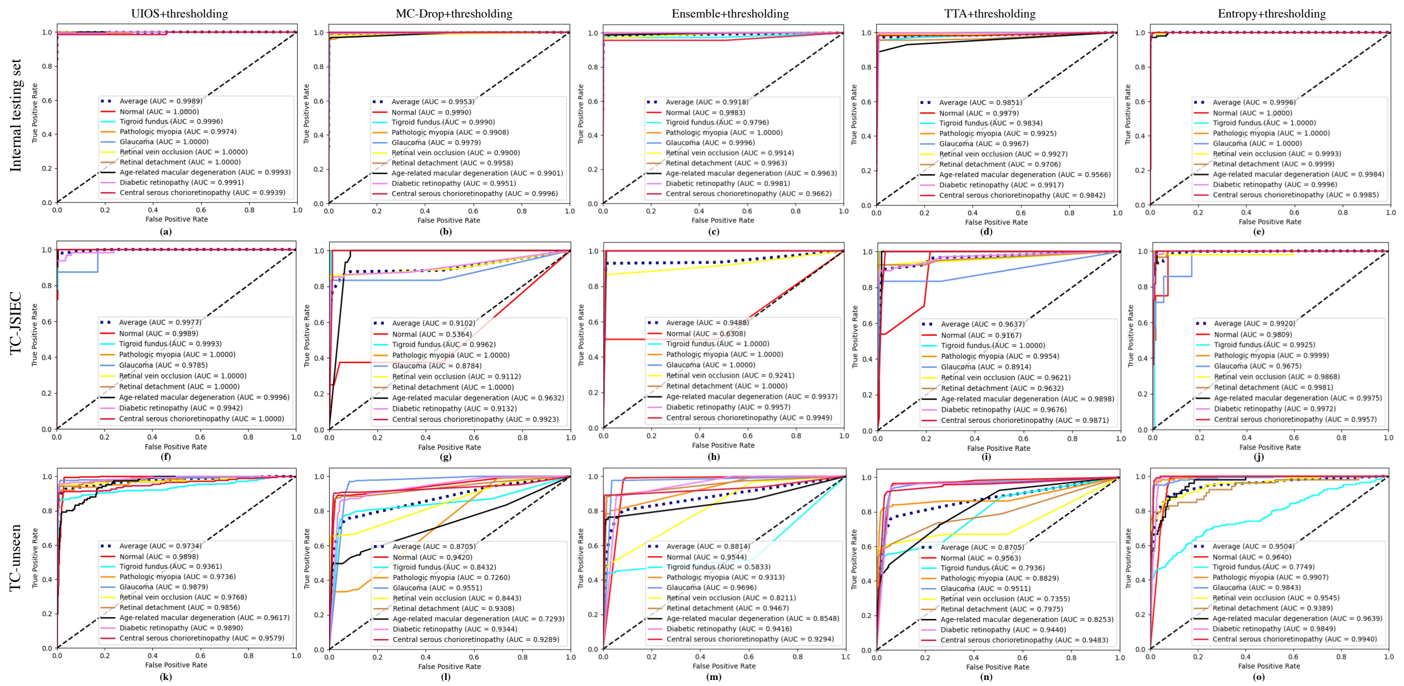

Supplementary Fig. 3. The receiver operating characteristic (ROC) curves of our UIOS+thresholding and other uncertainty-based methods+thresholding in internal and two external testing datasets. Source data are provided as a Source Data file.

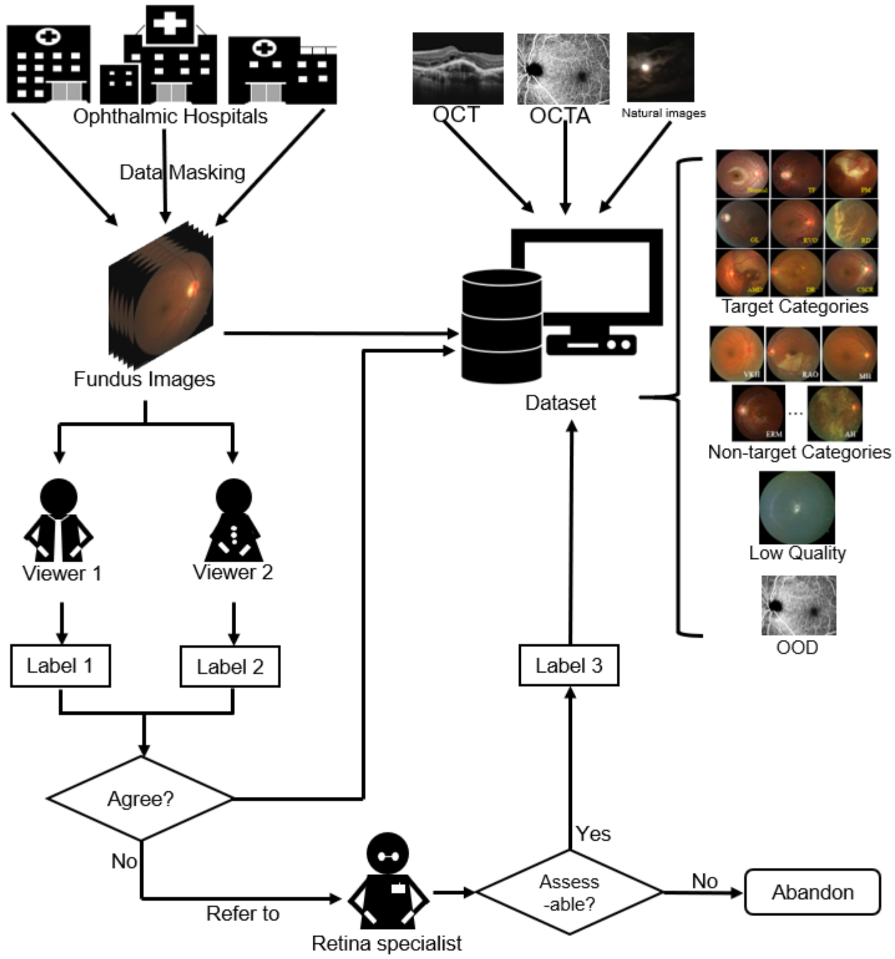

Supplementary Fig. 4. Flowchart of the data collection and annotation
