# Supplementary material for: Uncertainty-inspired open set learning for retinal anomaly identification
Source: Nat Commun. 2023 Oct 24;14:6757. doi: 10.1038/s41467-023-42444-7 (PMC10598011; doi:10.1038/s41467-023-42444-7)
Supplement: Supplementary file 3 — Reporting Summary [file 41467_2023_42444_MOESM3_ESM.pdf]

nature portfolio

Corresponding author(s): DAPR, NCOMMS-23-15702A

Last updated by author(s): Aug 23, 2023

Reporting Summary

Nature Portfolio wishes to improve the reproducibility of the work that we publish. This form provides structure for consistency and transparency in reporting. For further information on Nature Portfolio policies, see our Editorial Policies and the Editorial Policy Checklist.

Statistics

For all statistical analyses, confirm that the following items are present in the figure legend, table legend, main text, or Methods section.

n/a	Confirmed	
☐	☒	The exact sample size (*n*) for each experimental group/condition, given as a discrete number and unit of measurement
☐	☒	A statement on whether measurements were taken from distinct samples or whether the same sample was measured repeatedly
☐	☒	The statistical test(s) used AND whether they are one- or two-sided<br>*Only common tests should be described solely by name; describe more complex techniques in the Methods section.*
☒	☐	A description of all covariates tested
☒	☐	A description of any assumptions or corrections, such as tests of normality and adjustment for multiple comparisons
☐	☒	A full description of the statistical parameters including central tendency (e.g. means) or other basic estimates (e.g. regression coefficient) AND variation (e.g. standard deviation) or associated estimates of uncertainty (e.g. confidence intervals)
☐	☒	For null hypothesis testing, the test statistic (e.g. *F*, *t*, *r*) with confidence intervals, effect sizes, degrees of freedom and *P* value noted<br>*Give P values as exact values whenever suitable.*
☒	☐	For Bayesian analysis, information on the choice of priors and Markov chain Monte Carlo settings
☒	☐	For hierarchical and complex designs, identification of the appropriate level for tests and full reporting of outcomes
☒	☐	Estimates of effect sizes (e.g. Cohen's *d*, Pearson's *r*), indicating how they were calculated

Our web collection on statistics for biologists contains articles on many of the points above.

Software and code

Policy information about availability of computer code

Data collection	No software was used for data collection.
Data analysis	We trained our UIOS and other comparison methods on the public platform Pytorch and Nvidia Geforce RTX 3090 GPU (24G). Adam was adopted as the optimizer to optimize the model. The network implementation used the PyTorch deep learning framework (PyTorch 1.7.1).Result analyses are performed with custom code written in Python (python 3.9.13) with several packages, including numpy (numpy 1.21.5), and sklearn (sklearn 1.0.2). All codes are available at (https://github.com/LooKing9218/UIOS)

For manuscripts utilizing custom algorithms or software that are central to the research but not yet described in published literature, software must be made available to editors and reviewers. We strongly encourage code deposition in a community repository (e.g. GitHub). See the Nature Portfolio guidelines for submitting code & software for further information.

Data

Policy information about availability of data

All manuscripts must include a data availability statement. This statement should provide the following information, where applicable:

- Accession codes, unique identifiers, or web links for publicly available datasets
- A description of any restrictions on data availability
- For clinical datasets or third party data, please ensure that the statement adheres to our policy

Data from JSIEC1000 is available at (https://www.kaggle.com/datasets/linchundan/fundusimage1000).

Data from RETOUCH is available at (https://retouch.grand-challenge.org).
Data from VOC2012 is available at (http://host.robots.ox.ac.uk/pascal/VOC/voc2012).
Additional data sets supporting the findings of this study were not publicly available due to the confidential policy of National Health Commission of China, but are available from the corresponding authors upon reasonable request.

Research involving human participants, their data, or biological material

Policy information about studies with human participants or human data. See also policy information about sex, gender (identity/presentation), and sexual orientation and race, ethnicity and racism.

Reporting on sex and gender	N/A
Reporting on race, ethnicity, or other socially relevant groupings	N/A
Population characteristics	N/A
Recruitment	N/A
Ethics oversight	N/A

Note that full information on the approval of the study protocol must also be provided in the manuscript.

Field-specific reporting

Please select the one below that is the best fit for your research. If you are not sure, read the appropriate sections before making your selection.

☒ Life sciences ☐ Behavioural & social sciences ☐ Ecological, evolutionary & environmental sciences

For a reference copy of the document with all sections, see nature.com/documents/nr-reporting-summary-flat.pdf

Life sciences study design

All studies must disclose on these points even when the disclosure is negative.

Sample size	We collected 10,034 fundus images of 8 different fundus diseases or normal condition. Two non-target categories retinal diseases datasets(NTC dataset with 1,380 samples and NTC-JSIEC dataset with 502 samples) and one low quality image dataset with 1,066 samples were used to investigate the capability of UIOS to detect fundus abnormalities outside the categories of the training set. Three non-fundus photo public datasets were used to evaluate the performance of AI models in detecting OOD samples. The first was the VOC2012 dataset, with 17,125 natural images of 21 categories. The second was RETOUCH dataset which consisted of 6,936 2-D retinal optical coherence tomography images. The third was our OCTA dataset collected from our eye clinic, consisting of 304 2D retinal OCTA images. The sample size for training, validation and test was informed by the existing literature and works in the field of deep learning on fundus retinal images and Uncertainty study(Dolezal et al., 2022; Engelmann et al., 2022).
Data exclusions	The data collection and labelling procedure are shown in Supplementary Fig. 4. Fundus images from 5 eye clinics with different models of fundus cameras were collected. Two trained graders performed the annotation independently. If their results were inconsistent, a retinal sub-specialist with more than 10 years experience would make the final decision. Those images do not belong to any criteria mentioned in (Supplementary table.16) were considered unclassifiable and excluded. Moreover, we removed the images in the categories of massive hard exudate, cotton-wool spots, preretinal hemorrhage, fibrosis and laser spots from JSIEC to avoid confusion as reviewer suggested.
Replication	The code used for training the deep-learning models are made publicly available for the reproducibility purpose. Statistical analysis has been given as well. Specifically, we trained our UIOS and other comparison methods including standard AI model, Monte-Carlo drop-out (MC-Drop), ensemble models, time-augmentations (TTA), using entropy across the categorical class probabilities (Entropy), on the public platform Pytorch and Nvidia Geforce RTX 3090 GPU (24G). Adam was adopted as the optimizer to optimize the model. Its initial learning rate and weight decay were set to 0.0001 and 0.0001, respectively. The batch size was set to 64. To improve the computational efficiency of the model, we resized the image to 256×256.Meanwhile, online random left-right flipping was applied for data augmentation. All code is available at (https://github.com/LooKing9218/UIOS) for replication of all experimental results.
Randomization	We collected 10,034 fundus images of 8 different fundus diseases or normal condition. They were named the primary target-categories (TC) dataset. These images were randomly divided into training (6,016), validation (2,008) and test sets (2,010) in the ratio of 6:2:2.
Blinding	Image capture was based on daily practice of multi-centers which did not require blinding. Annotators for image labelling were blinded to the ground truth and were not involved in image collection. Engineers did not inspect the contents of datasets in algorithm developing and were blinded to the effect evaluation.

Reporting for specific materials, systems and methods

We require information from authors about some types of materials, experimental systems and methods used in many studies. Here, indicate whether each material, system or method listed is relevant to your study. If you are not sure if a list item applies to your research, read the appropriate section before selecting a response.

Materials & experimental systems

n/a	Involved in the study
☒	☐ Antibodies
☒	☐ Eukaryotic cell lines
☒	☐ Palaeontology and archaeology
☒	☐ Animals and other organisms
☒	☐ Clinical data
☒	☐ Dual use research of concern
☒	☐ Plants

Methods

n/a	Involved in the study
☒	☐ ChIP-seq
☒	☐ Flow cytometry
☒	☐ MRI-based neuroimaging
